# A Qualitative Study of Japanese Medical Students’ Perspectives on Clinical Practicum during Coronavirus Disease 2019

**DOI:** 10.31662/jmaj.2025-0087

**Published:** 2025-08-22

**Authors:** Tomoya Suzuki, Natsuya Sakata, Akihiko Ozaki, Tetsuya Tanimo, Yasushi Miyata, Yasuhiro Kotera, Elaina Taylor

**Affiliations:** 1 Department of Medicine, Okinawa Chubu Hospital, Okinawa, Japan; 2 Medical Governance Research Institute, Tokyo, Japan; 3 School of Medicine, Akita University, Akita, Japan; 4 Breast and Thyroid Center, Jyoban Hospital of Tokiwa Foundation, Fukushima, Japan; 5 Department of Internal Medicine, Navitas Clinic, Kawasaki, Kanagawa, Japan; 6 Department of Primary Care and Community Health, Aichi Medical University School of Medicine, Aichi, Japan; 7 Faculty of Medicine and Health Sciences, University of Nottingham, Nottingham, United Kingdom; 8 Center for Infectious Disease Education and Research, The University of Osaka, Suita, Japan; 9 College of Health, Psychology and Social Care, University of Derby, Derby, United Kingdom

**Keywords:** COVID-19, medical students, clinical practicums, online practicums, social contribution

## Abstract

**Introduction:**

Medical students are expected to contribute to society by applying their clinical knowledge and skills, particularly during times of crisis such as a pandemic. However, during the coronavirus disease 2019 pandemic, Japanese medical students faced strict restrictions on clinical involvement, which limited both educational opportunities and their ability to contribute. This study aimed to explore Japanese medical students’ experiences of clinical training under these conditions and to investigate their awareness of social contribution.

**Methods:**

This qualitative study involved semi-structured, peer-to-peer online interviews with 21 medical students from 19 universities across Japan. All participants had commenced hospital-based clinical training by September 1, 2022. Interviews were conducted between August and September 2022. Verbatim transcripts were analyzed using inductive thematic analysis.

**Results:**

Three main themes were developed: (1) Commitment to supporting patients and healthcare teams; (2) Decline in direct clinical learning opportunities; and (3) Online practicum: balancing benefits and drawbacks. While many students were eager to contribute, legal uncertainty, lack of practical training, and concerns about how patients perceived them acted as psychological and institutional barriers. At the same time, students emphasized the value of in-person clinical experience and demonstrated a growing awareness of professional identity.

**Conclusions:**

Despite limited clinical opportunities, Japanese medical students deepened their sense of professional responsibility and desire for social contribution during the pandemic. Medical education should provide clearer role definitions, institutional support, and hybrid models incorporating hands-on training to prepare students for future healthcare emergencies.

## Introduction

Medical students acquire clinical competencies and develop professional ethics through clinical clerkships ^(1), (2)^. At the same time, they are expected to contribute to society by applying the knowledge and skills they have gained. Social contribution by medical students plays an important role in fostering a sense of professional responsibility and the formation of a professional identity ^(3)^. Especially in times of crisis, such as pandemics, these activities are regarded not only as learning opportunities but also as valuable resources to overcome the crisis ^(4)^. However, the scope of medical students’ involvement in clinical care, including the provision of medical services, has raised ethical and legal concerns ^(5)^.

The role of medical students in such emergencies received considerable attention during the coronavirus disease 2019 (COVID-19) pandemic ^(6)^. Amid widespread disruption to medical education worldwide ^(7), (8)^, many universities temporarily suspended clinical training ^(9), (10), (11), (12)^. Nonetheless, in some Western countries, clinical clerkships were resumed under infection control measures, and students were given opportunities to participate in patient care and support activities related to COVID-19 ^(13), (14), (15)^. Though these were offered as opportunities to gain practical learning, some students experienced heightened anxiety and stress regarding their involvement due to a lack of guidelines and support ^(16)^.

In Japan, the resumption of clinical training was significantly delayed, and opportunities for medical students to participate in clinical care were highly restricted ^(17)^. In this context, it is important to explore how students perceived the changes in their clinical training and how they viewed their potential contribution to society. While prior studies have mainly focused on the loss of learning opportunities and psychological stress ^(16), (18), (19)^, few have examined Japanese students’ willingness to contribute and the ethical and psychological conflicts underlying their motivation.

This study aims to clarify (1) how Japanese medical students perceived the changes and restrictions in their clinical training during the pandemic, and (2) how they experienced and navigated their awareness of social contribution and the associated inner conflicts. To achieve this, we conducted peer-to-peer interviews to enable a deeper understanding of their perspectives.

## Materials and Methods

The methods were reported based on Consolidated Criteria for Reporting Qualitative Research guidelines ^(20)^.

### Design

We employed a qualitative approach to explore medical students’ experiences and needs. Unlike quantitative research, it allows for a diverse range of perspectives through individual narratives. Additionally, adopting the perspective of social constructionism ^(21)^, which views experiences as shaped by social contexts, not only enabled us to examine medical students’ experiences as personal events but also contributed to understanding their perspectives on clinical training within the broader societal context of the COVID-19 pandemic.

### Participants

The eligibility criteria included Japanese medical students over the age of 18, enrolled in medical departments of universities in Japan, and who had commenced hospital practicums as of September 1, 2022. The exclusion criteria were medical students who had not started their practicums. Recruitment was conducted using opportunity and snowball sampling. Participants were recruited in person by the primary researcher, who shared the invitation to the study through colleagues at several hospitals. These colleagues shared the invitation, along with a link to the study information, with medical students who were beginning clinical practicums. T.S. also invited direct acquaintances who were deemed suitable as subjects for this study. To reduce bias from pre-existing relationships, interviews were conducted neutrally, and the analysis was shared with a second researcher for objectivity. Participants who understood the purpose of the study and agreed to participate after reading the informed consent form were contacted by e-mail and interviewed. We determined the adequacy of our sample size based on the concept of information power, which considers factors such as the relevance of participants to the research question, the quality of dialogue, and the depth of data obtained. While traditional saturation models have been critiqued, particularly by Braun and Clarke ^(22), (23)^, we used a pragmatic approach to assess when sufficient insight had been achieved to meet the aims of our study. Although our initial aim was approximately 20 participants, we ultimately interviewed 21 individuals and judged this number to be sufficient given the narrow and specific study aim, the richness of the interview content, and the relative homogeneity of the participant group.

### Procedure and materials

The interviews used semi-structured questioning. Interview questions were developed based on discussion among the research team and the gap in existing literature. The questions were specifically developed through team discussions with an expert in qualitative research (Y.K.) and an expert in medical education (Y.M.), to ensure content relevance (the full list is provided in Appendix A). Interviews were conducted online using Zoom (Zoom Video Communications, Inc.), between August and September 2022, by Suzuki. Participants were encouraged to speak freely when responding to the questions (Table 1). Interviews were recorded using Zoom recording software and transcribed in real time during the interview. During transcription, the first author typed participants’ responses in real time while sharing the text on screen, allowing participants to verify and clarify their responses as the interview progressed. Interview transcripts were checked following the interview. The duration of each interview ranged from 30 minutes to 1 hour.

**Table 1.** Examples of Interview Questions.

Examples of interview questions
How do you feel about the significance of clinical practicums?
What was the practicum situation like during the COVID-19 pandemic?
Please tell us how the university responded to changes in the practicum format during the COVID-19 pandemic.
Did you feel that there was anything you could do as a medical student regarding the medical situation during the COVID-19 pandemic?
Do you have any thoughts on the societal contributions of medical students during the COVID-19 pandemic, including such volunteering and assisting with vaccination efforts during the COVID-19 pandemic?

COVID-19: coronavirus disease 2019.

### Data analysis

Inductive thematic analysis was used to analyze the interview data. This analysis method helps to organize and identify patterns of meaning (i.e., themes) throughout a dataset in a systematic manner ^(24)^. Thematic analysis is particularly helpful for identifying meanings and understanding idiosyncratic experiences. Moreover, it is noteworthy that the main function of thematic analysis is distinct from merely identifying commonalities in the data; instead, it focuses on evaluating the importance and relevance to the research questions. The six steps suggested by Braun and Clarke ^(24)^ were followed. This process included familiarization with the interview data. Transcripts were read repeatedly to understand the entire dataset and gain initial interpretations and patterns, informing possible themes ^(24), (25)^.

To develop the initial themes, we systematically assigned descriptive labels to codes identified within the interview transcripts ^(26)^. The initial themes were developed from the labeled dataset while maintaining alignment with the overarching research questions. Next, the initial themes were reviewed to determine whether they accurately captured the relevant dataset ^(24)^. The final themes are listed in Appendix B.

### Trustworthiness

We evaluated trustworthiness through four established criteria: credibility, dependability, confirmability, and transferability. To ensure rigor, we followed the trustworthiness criteria outlined by Thomas and Magilvy ^(27)^, including maintaining an audit trail, employing two coders (Suzuki and Sakata), and engaging in reflexive discussions. Credibility was strengthened through researcher triangulation among four researchers (T.S., N.S., A.O., T.T., and E.T.) and prolonged engagement with the data. Dependability was supported by thorough documentation of the analytic process, including coding manuals, decision trails, and records of team discussions. Confirmability was ensured through collaborative coding and reflexive memo writing. Transferability was addressed by providing detailed descriptions of the study context (Japanese medical education during the COVID-19 pandemic) and participant characteristics (medical students participating in clinical practicums).

## Results

**Figure 1.**
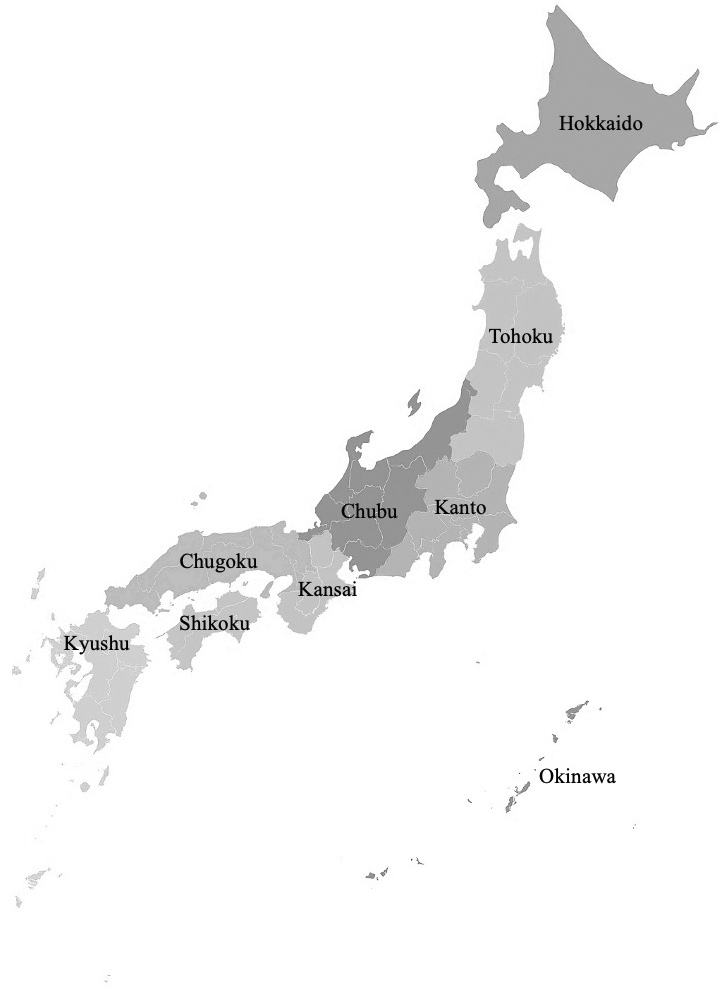
Japan Regional Map. This figure was created by the authors.

The study participants consisted of 11 males (52%) and 10 females (48%), with a median age of 23 years (IQR: 20-24). A total of 21 participants were recruited from seven different regional divisions in Japan, representing 19 universities. Table 1 presents the number of participants, along with their age, gender, academic year, and region of residence. Except for participants No. 6, No. 7, and No. 8, who were direct acquaintances, there was no pre-existing relationship between the interviewer and the participants. The average interview duration was 59 minutes (minimum 32 minutes, maximum 75 minutes, SD 10 minutes) (Figure 1 and Table 2)

**Table 2.** Demographic Details of Participants.

Number	Age	Sex	Grade	Area
1	23	Female	5	Kansai
2	23	Male	5	Hokkaido
3	24	Male	6	Chubu
4	23	Male	5	Tohoku
5	25	Female	6	Okinawa
6	22	Female	6	Chubu
7	22	Female	5	Tohoku
8	23	Male	5	Kanto
9	24	Male	6	Kanto
10	23	Female	5	Kyushu
11	23	Male	6	Kyushu
12	24	Female	6	Chubu
13	25	Male	6	Kanto
14	23	Male	5	Hokkaido
15	24	Female	6	Kansai
16	26	Male	6	Shikoku
17	25	Male	6	Kansai
18	24	Female	6	Kansai
19	25	Female	6	Sanin
20	25	Male	5	Kansai
21	22	Female	5	Kansai

Participant responses were developed into three final themes: (1) Commitment to supporting patients and healthcare teams, (2) Decline in direct clinical learning opportunities, and (3) Online practicum: balancing benefits and drawbacks. These themes are discussed in more detail below.

### Theme 1: Commitment to supporting patients and healthcare teams

There was a change in opportunities to engage in clinical practicums and face-to-face learning during the COVID-19 pandemic. This led some students to consider the motivation behind pursuing a career in medicine. There were many opinions expressing a desire to perform medical acts or administer COVID-19 vaccinations during the pandemic. For example, Participant 16 emphasized their willingness to contribute if allowed:

*I entered medical school because I wanted to perform medical acts, so if permitted, I would like to try. If it’s legally allowed, I would like to try. I think student doctors should be allowed to do it, so if the university permits, I would like to do more.*

Here, Participant 16 describes the importance of the practical application of the skills they have learned and their motivation to help and contribute to treating patients. Similarly, Participant 8 suggests that practical experience during the pandemic could lead to greater knowledge and preparedness in their careers as clinicians.

*During the COVID-19 pandemic, I was able to experience infection control and PCR testing. This experience was valuable because it would not have been possible without the COVID-19 outbreak. I believe that knowledge of this kind of practice will be useful in future medical activities.*

Some students felt a strong responsibility to help patients despite personal risks. During COVID-19, many wanted to support healthcare workers, recognizing the need for medical assistance. However, they were often uncertain about the legal limits of their role as student doctors. Participants 17 and 7 both expressed a willingness to contribute, such as by administering vaccines, but also raised concerns about the legal implications and responsibility in case of adverse events.

*If there is compensation, like in cases of medical negligence, I think it’s okay for medical students to administer vaccines.*

*If given the opportunity, I would like to try it. Regarding the COVID-19 vaccine, if issues such as side effects arise, though… if it’s pointed out that responsibility lies with medical students who don’t have a medical license, what should be done?*

Here, Participants 17 and 7 indicate a willingness to help but an uncertainty about the correct way to help, as well as concerns about professional liability. While some participants were keen to help, others felt worried about doing so, such as Participant 10, who felt that they were not ready to get involved:

*From the patient’s perspective, some see us as doctors because we’re student doctors, but they also see us as students. I also feel anxious about having blood drawn or being injected by my peers, so I would feel even more anxious performing this with regular patients.*

Similarly to Participant 10, some students were concerned about the opinions of others regarding receiving care from unqualified student doctors. Participants 4 and 13 expressed hesitations about performing medical procedures due to concerns about public trust, personal readiness, and patient anxiety:

*I feel the public opposes medical acts performed by students.*

*If asked to administer vaccines during COVID-19, I would feel a heavy responsibility. With my training canceled and no practice, I lack confidence. I also worry about managing patient anxiety and whether students can handle that.*

Participants were concerned about patient preferences and the responsibility of student doctors. They felt patients may prefer experienced doctors over students. Overall, students were willing to take on more responsibility, especially during times of need, and supported clearer guidelines on support and professional indemnity.

### Theme 2: Decline in direct clinical learning opportunities

Participants 17, 8, and 16 valued direct interaction with patients and tutors for fostering involvement, improving communication, and supporting hands-on learning.

*Unlike lectures, having patients present and being able to interact directly adds significance and makes me feel more involved.*

*Learning how to communicate with people is valuable. It’s enlightening to witness discussions among teachers.*

*I learned procedural skills. I immersed myself in orthopedic surgery from morning till night. Being called upon by teachers for guidance up close was invaluable. For those who prefer hands-on activities over mental exercises, practicums might be ideal.*

Participants 3 and 19 described issues in face-to-face practicums related to poor communication, unclear scheduling, and unwelcoming environments:

*Depending on the department, there were places where the intimidation was unjustified. During surgeries, if the atmosphere was unpleasant or if the progress of the operation was poor, some departments treated students rudely.*

*In face-to-face situations, the waiting time is long, and because the teacher is a doctor, I don’t know their schedule. No one tells me, but there was a lot of waiting.*

Participants noted that COVID-19 restrictions on clinical practicums varied by institution and department, often leading to limited patient contact or full suspension. Participant 15 described repeated interruptions due to positive cases, while Participant 6 noted that close contacts were also required to isolate:

*If a student tested positive for COVID-19, clinical training was suspended. The longest suspension occurred in May of the fifth year, during which all activities were conducted online.*

*If someone tested positive or was identified as a close contact, they were required to stay at home.*

All participants noted a shift to online practicums during COVID-19. While the pandemic restricted learning and patient access, pre-existing issues in face-to-face training, such as poor communication and scheduling, were also highlighted. These challenges indicate the need for curriculum improvements beyond the pandemic.

### Theme 3: Online practicum: Balancing benefits and drawbacks

Participants 4, 17, and 20 highlighted the benefits of online practicums, particularly in terms of time flexibility and balancing other responsibilities. Participant 4 noted:

*I do not have to commute to the university hospital. It’s easy and I can relax.*

Participant 17 appreciated the ability to manage time more freely:

*The advantage of online learning is the flexibility of time management and scheduling. Being at home makes it convenient.*

Participant 20 described how online classes supported classmates with parenting duties:

*I have a classmate who is a mother of one child, and she seems to be busy with taking her child to elementary school, so I think being able to spend time online is an advantage.*

Participant 15 noted that tutors used virtual tours to simulate the clinical environment:

*While face-to-face training was canceled, some teachers held online tours of their wards and testing departments to give us the clinical environment.*

While online practicums offered flexibility, participants noted several limitations in their implementation. In some cases, sessions were canceled or poorly executed due to tutors’ heavy workloads. Participant 11 shared:

*In the surgery department, a thread tying set was delivered to the students’ homes, and a tying lesson was planned. But the instructor was busy, and the lesson was not held.*

Participant 14 noted assumptions about students having materials for hands-on practice, which hindered participation:

*There was instruction on suturing practice online. We prepared just scissors and tweezers at home and [they] showed us how to hold them online. I couldn’t sew because I didn’t have a needle and thread.*

Participant 9 expressed frustration with passive learning and a lack of engagement:

*If unnecessary waiting time can be avoided, it’s better [by moving online]. But just watching surgeries gets boring. It’s my fault for not studying enough, but I don’t understand the difficult discussions the teachers are having.*

Participant 14 also highlighted a shift from practical training to lecture-based formats:

*During face-to-face training, participants practiced suturing, urinary catheter insertion, and CPR with detailed guidance. After moving online, the tutor demonstrated suturing tools over Zoom, but CPR training was reduced to a lecture.*

These comments reflect the limitations of online practicums, especially for skill-based training. While students appreciated the flexibility of online formats, they also emphasized the irreplaceable value of in-person practice for gaining confidence, motivation, and practical skills.

## Discussion

This study explored the clinical training experiences of Japanese medical students during the COVID-19 pandemic and their perspectives on contributing to healthcare and society. Three main themes were identified, among which Theme 1, “Commitment to supporting patients and healthcare teams,” was particularly distinctive and represents the most original contribution of this study. This theme offers novel insights into how students internalized professional values in a time of crisis, providing implications for future curriculum development and ethical training in medical education.

One notable finding was that students exhibited a strong intrinsic motivation to “contribute to society” (Theme 1) in the unprecedented context of a global pandemic. This awareness extended beyond the role of a passive learner and can be interpreted as an early manifestation of professional responsibility and identity as future physicians.

### Ethical motivation and willingness to act

Students expressed a desire to support healthcare efforts despite recognizing their own inexperience and the risk of infection. Their reflections on fundamental questions―such as why they chose to pursue medicine―suggest a deep engagement in the process of professional self-formation. Similar findings have been reported in international studies, which highlight professional responsibility and altruism as key ethical motivators behind medical students’ willingness to act during the COVID-19 crisis ^(28), (29), (30)^.

### Legal and institutional barriers

At the same time, many students hesitated to act due to uncertainties about legal liability and the risks associated with performing clinical tasks without a license. The lack of clear guidelines and institutional support served as significant barriers to translating motivation into action. Similar concerns have been documented in other countries, where the presence or absence of regulatory frameworks strongly influenced the feasibility of student involvement ^(31)^.

### Lack of confidence and anxiety about patients’ perceptions

Additionally, students’ lack of confidence stemming from inadequate training, along with anxiety about how they would be perceived by patients, posed further psychological obstacles. These concerns are closely linked to self-efficacy and the formation of professional identity ^(16)^ and represent essential yet challenging aspects of the professional development process in medical education.

### From limited clinical exposure to heightened responsibility: The interplay of themes 2, 3, and 1

Theme 2 (Decline in direct clinical learning opportunities) revealed that the suspension of face-to-face practicums during the pandemic significantly hindered students’ ability to develop procedural skills and engage with patients, a challenge similarly noted in other studies on undergraduate medical education during COVID-19 ^(32)^. This reduction in hands-on training disrupted both skill acquisition and opportunities to observe real-world medical practice, as shown in previous studies ^(33), (34), (35)^. In addition, students highlighted pre-existing issues such as vague schedules and limited supervision, pointing to the need for systemic reform in clinical education.

Theme 3 (Online practicum: balancing benefits and drawbacks) showed that although online learning offered flexibility and infection control, it fell short in fostering clinical realism and active participation. Students reported difficulty in feeling a sense of role ownership and professional presence in the virtual environment ^(36), (37)^.

These educational constraints―particularly the lack of real clinical exposure―provided students with opportunities to reflect on their roles―prompting questions such as: “What can I do in this situation?” or “How can I contribute to society as a future physician?” The absence of in-person clinical practicums and the transition to online learning heightened students’ motivation to contribute, thereby strengthening their sense of social responsibility and professional identity.

### Future directions

Although medical students showed a strong desire to contribute during the pandemic, limited institutional and psychological support in Japan hindered their involvement. In contrast, the UK and US established systems that enabled supervised student participation, fostering both skills and professional identity. To support similar engagement in Japan, clearer role definitions, structured guidelines, and educational support are needed. Future reforms could include structured volunteer roles for students in public health efforts during emergencies, supervised by licensed professionals. Hybrid learning and psychological support may also help cultivate clinical competence and ethical motivation in future medical education.

### Limitations

A key strength of this study is that it included a range of participants across universities in Japan. The research findings are therefore more likely to represent a wider range of experiences and may be more applicable to general experiences of medical students in Japan. However, this study focused only on Japanese medical students and did not include other healthcare professions. As a result, the findings may not fully reflect interprofessional dynamics during the pandemic. Findings may not be generalizable to all students due to the qualitative design.

### Conclusions

Japanese medical students developed a stronger sense of social contribution and professional identity during the COVID-19 pandemic, despite limited clinical opportunities. These constraints prompted deeper reflection on their roles as future physicians. Clearer institutional support is needed to foster student engagement in similar situations. Supporting this motivation through clear institutional frameworks may be key to fostering resilient and socially responsible future physicians.

## Article Information

### Conflicts of Interest

Tetsuya Tanimo reports personal fees from Medical Network Systems, MNES Inc., and Bionics co. ltd., outside the submitted work. Akihiko Ozaki reports personal fees from Medical Network Systems, MNES Inc., outside the submitted work. All remaining authors have nothing to disclose.

### Author Contributions

Substantial contributions to the conception and design of the study: Tomoya Suzuki. Conducted all interviews, performed data analysis, drafted the manuscript, and approved the final version: Natsuya Sakata. Contributed to data analysis and critically revised the manuscript for important intellectual content; Akihiko Ozaki. Supervised the study design and provided substantial input into manuscript revision: Tetsuya Tanimo. Contributed to project oversight and critical revision of the manuscript: Yasushi Miyata, and Yasuhiro Kotera. Contributed to the study design and provided methodological expertise and manuscript feedback: Elaina Taylor. Contributed to the theoretical framing of the study, advised on qualitative methods, and critically revised the manuscript. All authors reviewed and approved the final version of the manuscript and agreed to be accountable for all aspects of the work.

### Approval by Institutional Review Board (IRB)

This study was reviewed and approved through an expedited review process by the Ethics Review Committee of the Medical Governance Research Institute on August 24, 2022, following a protocol amendment submitted on August 1, 2022 (Approval Number MG2022-02).

## Supplement

Appendix
