# Supplementary material for: A Qualitative Study of Japanese Medical Students’ Perspectives on Clinical Practicum during Coronavirus Disease 2019
Source: JMA J. 2025 Aug 22;8(4):1132–40. doi: 10.31662/jmaj.2025-0087 (PMC12598261; doi:10.31662/jmaj.2025-0087)
Supplement: Appendix [file 2433-3298-8-4-1132-s001.pdf]

Appendix A

RQ1: How Japanese medical students perceived the changes and restrictions in their clinical training during the pandemic.

・How do you feel about the significance of clinical practicums?

・If you have experience with face-to-face practicums, please tell us about the advantages and disadvantages of face-to-face practicums and their solutions.

・If you have encountered online practicums, please tell us about the advantages and disadvantages, and their solutions.

・Please tell us about the most significant aspect of the practicum you have experienced (including face-to-face and online).

・What was the practicum situation like during the COVID-19 pandemic? Please tell us about the situation regarding the suspension of practicums and the transition to online education.

・If there were suspensions, how long did they last? What were the alternative measures?

・In the case of online education, what specific activities were conducted?

・In the case of restricted hospital practicums (e.g., no contact with patients, half-day sessions), please provide details.

・For practicums outside of the university, please tell us about the situation, such as suspensions or restrictions.

・Please tell us how the university responded to changes in the practicum format during the COVID-19 pandemic.

Follow-up questions:

・If practicums were suspended or transitioned online, what options were presented to students by the university?

・Was there any support provided to students who preferred face-to-face practicums (such as conditional face-to-face practicums)?

・If there was any support from the university, please provide specific examples of your experience.

RQ2: How they experienced and navigated their awareness of social contribution and the associated inner conflicts.

・Did you feel that there was anything you could do as a medical student regarding the medical situation during the COVID-19 pandemic?

・In Western countries, medical students have been volunteering and assisting with vaccination efforts during the COVID-19 pandemic. Additionally, during the peak of the pandemic, medical students were deployed to the frontline of medical care. Were you aware of such situations? What are your thoughts?

・Furthermore, do you have any thoughts on the societal contributions of medical students during the COVID-19 pandemic, including such activities?

・Why do you think such activities are not common in Japan?

・Do you think Japan should adopt such practices?

・Do you have any intentions to participate in such activities? If so, please provide your opinions on the advantages and risks involved.

Appendix B

Code generated (n=14)

Table: Initial codes and Final Themes

Initial codes	Final Themes
Responsibility for medical practice<br>Meaningful face-to-face training	Theme 1) Commitment to supporting patients and healthcare teams
Financial support<br>Mental support<br>Anxiety about medical procedures<br>Disadvantages of online training<br>Disadvantages of face-to-face training	Theme 2) Decline in direct clinical learning opportunities
Positivity toward medical treatment<br>Variation in responses by teachers<br>Individual treatment by clinical department<br>Contact with patients<br>Cancellation of off-campus training<br>Cancellation of training<br>Partially cancelled	Theme 3) Online practicum: balancing benefits and drawbacks
